# Electromechanical Impedance Model for Free 1D Thin-Walled Piezoelectric Ceramics with a Novel Derivation

**DOI:** 10.3390/ma13214735

**Published:** 2020-10-23

**Authors:** Xiangxing Kong, Chunyang Chen, Xiao Liu, Jianjian Zhu, Xinlin Qing

**Affiliations:** 1 Aero Engine Academy of China, Beijing 101304, China; aeac08@aeac.aecc.cn; 2 School of Aerospace Engineering, Xiamen University, Xiamen 361102, China; chenchunyang0517@stu.xmu.edu.cn (C.C.); liuxiao@stu.xmu.edu.cn (X.L.); xinlinqing@xmu.edu.cn (X.Q.)

**Keywords:** structural health monitoring, electromechanical impedance, piezoelectric ceramics, correlation coefficient

## Abstract

The electromechanical impedance model of the piezoelectric ceramics in a free state can be used for screening and quality control in the structural health monitoring community, but the derivation process of the existing model is usually complicated. This paper describes a novel theoretical derivation methodology based on the assumption of zero-stress on the free boundary of the one-dimensional transducer, which can simplify the derivation of the model to a large extent. To assess the accuracy of the model, a signal processing method based on frequency shifting transformation and the Pearson correlation coefficient is also proposed to calculate the similarity between theoretically predicted and experimentally measured data. Two different piezoelectric ceramics were used in experiments to verify the effectiveness of the model. Experimental results convincingly demonstrate that the assumption proposed in this paper possesses good feasibility for one-dimensional thin-walled piezoelectric ceramics and the model has excellent precision.

## 1. Introduction

Over the past decade, electromechanical impedance (EMI) based on piezoelectric ceramics (PZT) emerged as a cost-effective technique in structural health monitoring (SHM) and non-destructive testing (NDT) communities. The EMI technique has been widely used for damage detection in typical engineering structures, such as infrastructures, composites, and mechanical equipment.

At present, the SHM techniques are mainly based on the PZT sensor array to acquire the serving condition in real-time. Hence, the properties of PZT have a great influence on the monitoring accuracy, which may lead to poor detection results if the PZT used in the sensing network has a difference. The priori to the monitoring of structural condition with the EMI technique is the screening of PZT sensors before mounting on the host structures. It is significant to test and determine the performance of a PZT in its free state when excited by an alternating electric charge. For a better understanding of the PZT spectroscopy in its free state, a relevant theoretical model needs to be established to reveal the characteristics of rectangular PZT signals changing with excitation frequency in its longitudinal direction.

Research workers have made substantial efforts to the analytical and numerical modeling of various aspects in the EMI field up to date. Liang et al. [1] firstly proposed the EMI equations whose theoretical model aimed at a coupled “rectangular PZT-structure” application. Bhalla et al. [2,3,4] and Zhou et al. [5,6] subsequently modified the theoretical models for the square PZT, respectively. Annamdas et al. [7,8] proposed the three-dimensional EMI models via considering the interaction between square PZT and host structures. Giurgiutiu et al. [9] studied a theoretical model based on a circular piezoelectric transducer. Rajabi et al. [10] proposed a crack detection method in a rectangular plate on the basis of the EMI method with theoretical modeling and experimental verification. Damage metrics such as Root Mean Square Deviation (RMSD), Mean Absolute Percentage Deviation (MAPD), and Correlation Coefficient Deviation (CCD) are used in their investigation. The results showed that the generation of cracks and crack depths increasing can be detectable by CCD.

Apart from the above-mentioned researchers who proposed their physical models of EMI, there is also much other work reported by scholars. The scope of these investigations covered structural identification and damage diagnosis, signal processing, and damage localization. For instance, Zhang et al. [11] proposed an EMI-based method to monitor the stiffness degradation of the hardened cement paste under uniaxial compression loading. Their research well validates the effectiveness and applicability of the EMI method in real-time monitoring of cement stiffness degradation under compression loadings. Silva et al. [12] applied the EMI technique in the monitoring of sigma phase embrittlement in duplex stainless steel. This method is considered as a nondestructive test, presents accurate results with high sensitivity and reliability providing a capacity of determining sigma phase embrittlement, and has promising application potential in this and other materials. Hamzeloo et al. [13] developed a method based on EMI to detect the damage of the L-Shaped beam structure with a crack. The results showed that the EMI responses of the structure depend on excitation and natural frequencies of the structure which emerge as resonance frequencies in the impedance spectrum and demonstrates the efficacy of predicted spectra. Zhu and Qing et al. [14] reported a damage detection and imaging algorithm based on the EMI technique. They used the EMI technique to locate the debonding inside the honeycomb sandwich composite structure. To display the debonding intuitively, a modified imaging algorithm developed from a probability-weighted principle was presented. Tawie et al. [15] studied the damage detection performance of the EMI technique with various attachment methods on glass fiber composite plates. The performance of each attachment method for identifying different damage types has been analyzed and finite element analysis (FEA) was carried out for verification of the experimental results. Fiborek et al. [16] presented a time-domain spectral element method for modeling the EMI of debonding composites. The investigation comprised of modeling using the spectral element method and experimental measurements. Numerical and experimental spectra were analyzed. Differences in spectra caused by differences in considered samples were observed. Wandowski et al. [17] studied the temperature and damage influence on the EMI method used for carbon fiber-reinforced polymer panels. The damage is in the form of artificially made delamination with different sizes. They also discussed the problem of the influence of the structure’s boundary condition on low-frequency measurements. Djemana et al. [18] used EMI and extreme learning machine (ELM) to detect and locate damage inside structures. The method was confirmed by experiments and the results showed that ELM can be used as a tool to predict a single instance of damage in structures. Martowicz et al. [19] applied the EMI technique for damage detection in a bolted pipeline connection. Their work discussed the effectiveness of the EMI-based SHM system for damage detection in a laboratory test on a bolted pipeline. Sahm et al. [20] used the EMI technique to develop a monitoring system to detect structural damage. The fatigue experiments were carried out and the results demonstrated the applicability of their method on real-life structures. Ai et al. [21] performed a comparative investigation on the mechanical impedance-based embedded piezoelectric transfer for reinforced concrete structural impact damage detection. Their experimental results demonstrated that the proposed ESMI (Effective Structural Mechanical Impedance) signatures exhibit good performance in detecting the Reinforced Concrete (RC) beams structural impact damage. Guo et al. [22] introduced a PZT-based SHM system for impact monitoring and impedance measurement. The paper presented an application of the new metric for the detection of damage in metallic and composite structures. The experimental results indicated that the proposed variable F increases the robustness of the damage detection as compared to the quantities R and G, which demonstrated the effectiveness of the methodology. Campos et al. [23] proposed a feature extraction approach insensitive to temperature variation for the impedance-based SHM technique. This study presents a new feature extraction approach that is insensitive to temperature variations. The experimental results indicated that the proposed method was capable of detecting and quantifying structural damage in environments under temperature variation. Dixit et al. [24] studied prognosis of fatigue and impact induced damage in concrete using embedded piezo-transducers. Based on these measurements, separate relationships have been developed for impact and fatigue induced damages linking the PZT-identified damping with remaining life. Providakis et al. [25] studied the detection of concrete reinforcement damage using piezoelectric materials with analytical and experimental methods. A series of experiments were designed and the results are very promising. Chalioris et al. [26] studied the experimental application of a wireless earthquake damage monitoring system (WiAMS) using PZT transducers in reinforced concrete beams. The experimental results showed that the use of PZTs for detecting earthquake damage in reinforced concrete structures by employing the electromechanical impedance approach can be considered as a highly promising non-destructive structural health monitoring method. Constantin et al. [27] studied the issue of SHM of seismically vulnerable RC frames under lateral cyclic loading. The effectiveness of a Wireless impedance/Admittance Monitoring System (WiAMS) for the prompt damage diagnosis of the Reinforced Concrete (RC) frames under cyclic loading is experimentally investigated. The test results highlighted the ability of the proposed SHM to identify incipient damages due to concrete cracking and steel yielding, promising early indication of forthcoming critical failures before any visible sign has been obtained.

However, the majority of previous investigations were mainly focused on the fundamentals of PZT-structure interaction, damage localization, and diagnosis in the coupled state. Only a few studies concentrated on impedance spectroscopy of PZT under free conditions. The spectroscopy of a free PZT can be used for determining the sensors’ selection and its layout for the subsequently coupled scenarios that integrate the PZT with monitored structures, such as the surface-mounted or embedded PZT. To sum up, it is significant to derive a model aiming at PZT in a free state to assess its performance.

In this paper, a novel derivation of the PZT theoretical model in a free state was described by considering the two assumptions: (1) zero-stress boundary condition, and (2) longitudinal vibration in 1-direction. Then the theoretical model for one-dimensional can be deduced. By verifying the proposed EMI model with experiments, the effectiveness and accuracy of the model can be convincingly demonstrated. Furthermore, the model can be used for predicting the PZT spectroscopy by inputting mechanical and electrical parameters, which has favorable guidance in promoting the monitoring results and improving the cost-effectiveness in the field of SHM.

## 2. Theoretical Derivation

**Figure 1:**
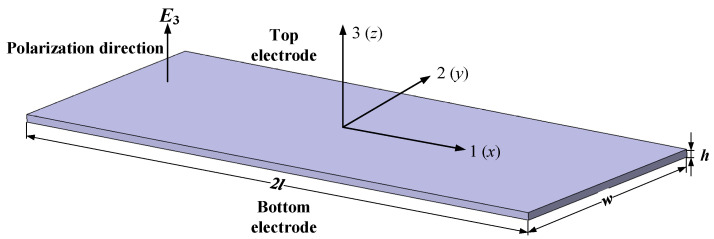
Schematic of a thin-wall 1D piezoelectric sensor.

A thin-walled rectangular PZT has a length of *2l*, width *w*, and thickness *h*. The PZT is assumed to undergo the longitudinal expansion along the 1-direction induced by the electric field *E*_3_ in thickness polarization, as shown in Figure 1. The electric field *E*_3_ is produced by applying the harmonic voltage between the top electrode and bottom electrode, which is defined by $V\left(t\right)=V_{0}e^{j\omega t}$. The electric field is assumed to be uniform over the whole thin-walled piezoelectric ceramics.

Defining the size in length, width, and thickness meets the correlation of 2 *l* >> *w* >> *h*, then the motions in three directions can be considered as uncoupled. Assuming the displacement in 1-direction is *u*_1_ and the vibration in a longitudinal direction (1-direction) is regarded as a predominant mode.

According to the above-mentioned assumption that the electric field *E*_3_ is uniform over the PZT, the related derivative is a constant zero, i.e., $\partial E_{3}/\partial x=0$. Due to the harmonic voltage excitation, the electric field then can be expressed with the plural form, i.e., $E_{3}=E_{3}e^{j\omega t}$. Hence, the displacement response in 1-direction is also harmonic, which is $u_{1}\left(x,t\right)=u_{1}\left(x\right)e^{j\omega t}$. The notation $u_{1}\left(x\right)$ represents the displacement in 1-direction.

**Figure 2:**
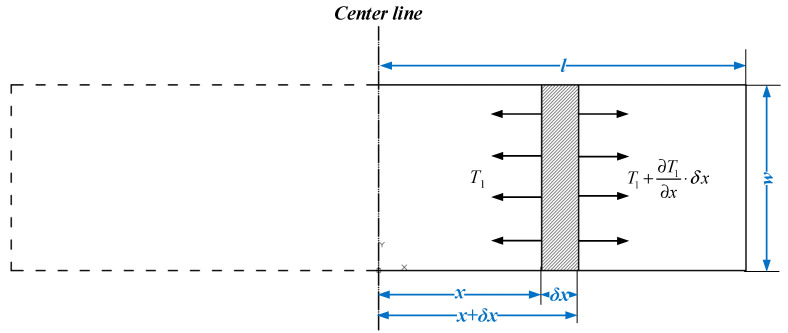
Infinitesimal element of piezoelectric ceramics (PZT).

Assuming that there is an infinitesimal element of the PZT and the length of the element is defined as δx, the element situates at a distance *x* from the centroid under dynamic equilibrium condition. The illustration of the infinitesimal element of the PZT is shown in Figure 2.

According to Figure 2, the mass of the infinitesimal element can be determined by Equation (1):

(1) dm=ρwhδx

where the notation ρ is the density of piezoelectric material. By applying the D’Alembert’s principle on the selected infinitesimal element, the Equation (2) can be obtained.

(2) $$\left[\left(T_{1}+\frac{\partial T_{1}}{\partial x}\times \delta x\right)-T_{1}\right]\times wh=dm\times \frac{\partial^{2}u_{1}\left(x,t\right)}{\partial t^{2}}$$

Substituting Equation (1) into Equation (2) and solving then yields Equation (3):

(3) $$\frac{\partial T_{1}}{\partial x}=\rho \frac{\partial^{2}u_{1}\left(x,t\right)}{\partial t^{2}}$$

According to Hooke’s Law and a correlation between displacement and strain, Equation (3) can be transformed into Equation (4):

(4) $$\frac{1}{s_{11}^{E}}\times \frac{\partial^{2}u_{1}\left(x,t\right)}{\partial x^{2}}=\rho \frac{\partial^{2}u_{1}\left(x,t\right)}{\partial t^{2}}$$

By solving the governing wave equation (partial differential equation (PDE)), as shown in Equation (4), and applying the initial value, i.e., *x =* 0, *u =* 0, the general solution of this PDE can be obtained, as shown in Equation (5):

(5) $$u_{1}\left(x,t\right)=A\sin\left(\kappa x\right)e^{j\omega t}$$

where the notation κ denotes the wavenumber, which is defined by Equation (6):

(6) $$\kappa =\omega \sqrt{\rho s_{11}^{E}}$$

Considering the thin-walled rectangular PZT vibrates longitudinally in 1-direction, the piezoelectric constitutive relations can be simplified, as described in Equation (7):

(7) $$\left[\begin{array}{l} S_{1}\\ D_{3} \end{array}\right]=\left[\begin{array}{cc} s_{11}^{E} & d_{31}\\ d_{31} & \varepsilon_{33}^{T} \end{array}\right]\left[\begin{array}{l} T_{1}\\ E_{3} \end{array}\right]$$

where *S*_1_ is the strain in a longitudinal direction (1-direction). *D*_3_ is the electric displacement of the PZT. *d*_31_ is the piezoelectric strain coefficient. *T*_1_ is the axial stress in 1-direction. $Y_{11}^{E}$ is Young’s modulus of elasticity of the piezoelectric ceramic. $\varepsilon_{33}^{T}$ is the dielectric constant of the PZT.

According to the Equation (7), the expression of *T*_1_ can be deduced as Equation (8):

(8) $$T_{1}=\frac{1}{s_{11}^{E}}\left(S_{1}-d_{31}E_{3}\right)$$

In the light of “displacement-strain” relation, the equation of *S*_1_ can be determined by Equation (9):

(9) $$S_{1}=\frac{\partial u_{1}\left(x,t\right)}{\partial x}=Ae^{j\omega t}\kappa \cos\left(\kappa x\right)$$

Substituting Equation (9) into Equation (8), then yields the expression of *T*_1_, as shown in Equation (10):

(10) $$T_{1}=\frac{1}{s_{11}^{E}}\left[Ae^{j\omega t}\kappa \cos\left(\kappa x\right)-d_{31}E_{3}\right]$$

Assuming that the thin-walled rectangular PZT is vibrating longitudinally, the boundary condition can be regarded as a “free” state, which means that the stress on the boundary of PZT is zero, as shown in Equation (11):

(11) $$T_{1}|{}_{x=l}=0$$

Substituting Equation (10) into Equation (11), the Equation (12) can be obtained. Then the expression of to-be-determined constant *A* can be given by Equation (13):

(12) $$Ae^{j\omega t}\kappa \cos\left(kl\right)-d_{31}E_{3}=0$$

(13) $$A=\frac{d_{31}E_{3}}{e^{j\omega t}\kappa \cos\left(\kappa l\right)}$$

Hence, Equation (5) can be further determined as the following Equation (14):

(14) $$u_{1}\left(x,t\right)=\frac{d_{31}E_{3}}{\kappa \cos\left(\kappa l\right)}\sin\left(\kappa x\right)$$

According to Equation (7), the electric displacement can be deduced as Equation (15):

(15) $$D_{3}=\frac{1}{s_{11}^{E}}d_{31}^{2}E_{3}\left[\frac{1}{\cos\left(kl\right)}\cos\left(\kappa x\right)-1\right]+\varepsilon_{33}^{T}E_{3}$$

Due to the correlation between the electric displacement and current, the expression of current over the PZT surface can be deduced as shown in Equation (16):

(16) $$I=\iint_{\Sigma }\frac{dD_{3}}{dt}dxdy=j\omega \iint_{\Sigma }D_{3}dxdy$$

where the notation Σ represents the integration field. Then the expression of current can be subsequently determined as Equation (17):

(17) $$I=w\times 2l\times j\omega \times \left(\varepsilon_{33}^{T}E_{3}-\frac{d_{31}^{2}E_{3}}{s_{11}^{E}}\right)+j\omega \times w\times \frac{d_{31}^{2}E_{3}}{s_{11}^{E}\cos\left(\kappa l\right)}\times \frac{2\sin\left(\kappa l\right)}{\kappa }$$

Considering the definition of the electric field, i.e., $E_{3}=V\left(t\right)/h$, then the expression of admittance and impedance can be deduced and further simplified, as shown in Equations (18) and (19):

(18) $$Y=j\omega \times \frac{w\cdot 2l}{h}\times \left[\varepsilon_{33}^{T}-\frac{d_{31}^{2}}{s_{11}^{E}}\left(1-\frac{\tan\left(\kappa l\right)}{\kappa l}\right)\right]$$

(19) $$Z=Y^{-1}=\frac{1}{j\omega }\times \frac{h}{w\cdot 2l}\times {\left[\varepsilon_{33}^{T}-\frac{d_{31}^{2}}{s_{11}^{E}}\left(1-\frac{\tan\left(\kappa l\right)}{\kappa l}\right)\right]}^{-1}$$

Practically, there are mechanical and dielectric losses when the PZT is excited by the harmonic voltage. Thus the mechanical loss factor *η* and dielectric loss factor *δ* are separately defined. The Young’s modulus and dielectric coefficient taking the loss effect into account can be expressed by Equations (20) and (21):

(20) $$\bar{s_{11}^{E}}=s_{11}^{E}\left(1-\eta j\right)$$

(21) $$\bar{\varepsilon_{33}^{T}}=\varepsilon_{33}^{T}\left(1-\delta j\right)$$

Then the formulae of admittance and impedance can be further transformed into the following complex expressions, as shown in Equations (22) and (23):

(22) $$\bar{Y}=j\omega \times \frac{w\times 2l}{h}\times \left[\bar{\varepsilon_{33}^{T}}-\frac{d_{31}^{2}}{\bar{s_{11}^{E}}}\left(1-\frac{\tan\left(\bar{\kappa }l\right)}{\bar{\kappa }l}\right)\right]$$

(23) $$\bar{Z}=\frac{1}{j\omega }\times \frac{h}{w\times 2l}\times {\left[\bar{\varepsilon_{33}^{T}}-\frac{d_{31}^{2}}{\bar{s_{11}^{E}}}\left(1-\frac{\tan\left(\bar{\kappa }l\right)}{\bar{\kappa }l}\right)\right]}^{-1}$$

The Equations (22) and (23) are practically formulae of the PZT under the free conditions in a longitudinal direction. Compared with the existing model, the derivation process is much simpler based on zero-stress assumption on the PZT boundary presented in this paper.

## 3. Experimental Setup

To demonstrate the effectiveness and applicability of the formulae proposed in this paper, two verifying experiments were designed and conducted. In addition, to simulate the free state of PZT, a simple fixture was also machined and used for clamping the centroid of PZT.

**Figure 3:**
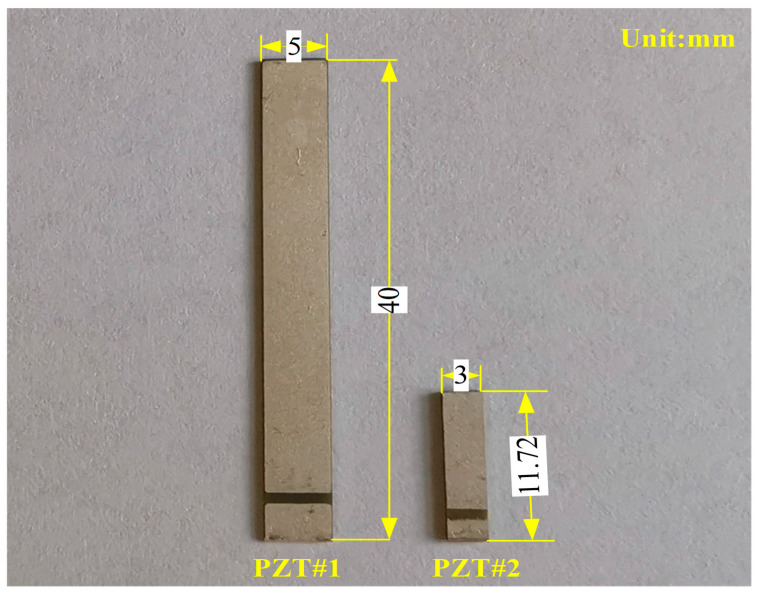
Comparative schematic of PZT#1 and #2.

For convincingly demonstrating the predicted curve with the EMI model presented in the theoretical section, two different PZTs were applied in the experiments. The thin-walled rectangular PZTs used in experiments were provided by the Shanghai Institute of Ceramics, PZT-5A (numbered PZT#1) and PZT-5A1 (numbered PZT#2). The size of PZT-5A and PZT-5A1 are separately 40 mm × 5 mm × 0.34 mm and 11.72 mm × 3 mm × 0.78 mm in length, width, and thickness direction. The properties of PZT with electrodes coated silver on both sides are enumerated in Table 1. The comparative schematic of the two mentioned PZTs used in experiments is illustrated in Figure 3.

**Figure 4:**
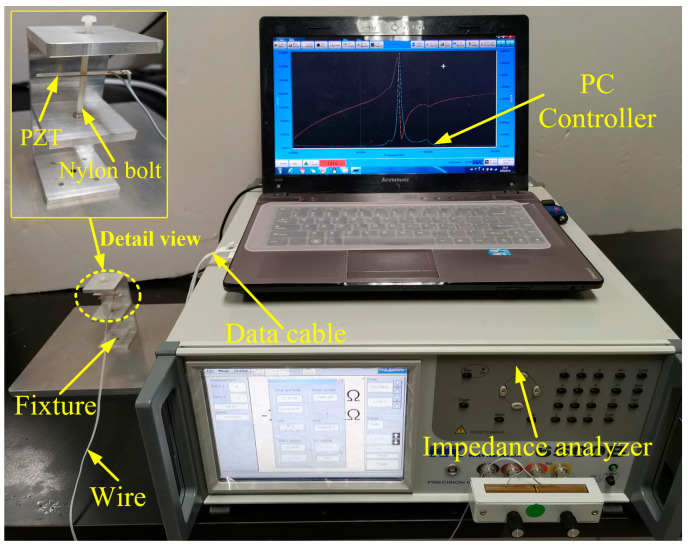
The testing platform of PZT in a free state.

The fixture used for clamping the PZT was made of 6061-T6 aluminum alloy. Two nylon bolts were adopted to constrain the PZT to prevent the top and bottom electrodes from short-circuiting, as shown in Figure 4. A cable was employed to connect the laptop with the impedance analyzer to transmit data. Meanwhile, a conductive wire was soldered with PZT and connected with an impedance analyzer to acquire experimental signals.

During the experiments, the resistance and reactance signals were acquired in terms of the above-mentioned experimental setup. Owing to the discrepancy among PZTs in materials and sizes, there are also some differences in their sampling frequency scopes. Because the PZTs under free condition are easily excited, the frequency scope of the main peak was also considered. The interference was also found in our laboratory when the frequency was less than 10 kHz. Therefore, the testing frequency scope from 10 kHz to 200 kHz was finally selected. More precisely, as to PZT-5A (numbered PZT#1), the scope of frequency ranges from 10 k–200 kHz, while the PZT-5A1 (numbered PZT#2) ranges from 50 k–200 kHz. The data points are 800 in each experiment, which means that the uniform sample interval is 237.5 Hz and 187.5 Hz, respectively.

## 4. Results and Discussion

### 4.1. Comparison between Theoretical and Experimental Data of PZT#1

Based on the Equation (23) and the properties enumerated in Table 1, the real part (resistance, *R*) and imaginary part (reactance, *X*) of the impedance of PZT under free condition can be calculated.

**Figure 5:**
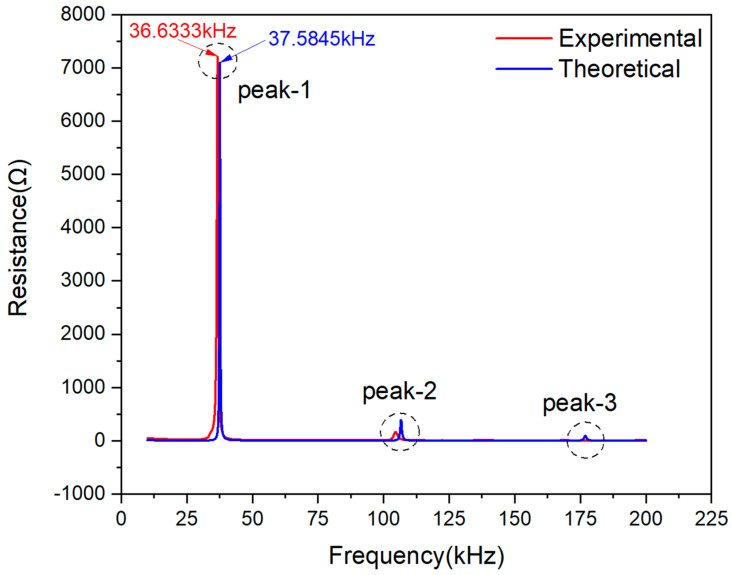
Experimental and theoretical resistance plots comparison of PZT#1.

As mentioned above, to demonstrate the model’s effectiveness in this paper, two kinds of piezoelectric ceramics, PZT#1 and #2, were adopted. The experimental and theoretical curve of PZT#1 determined by Equation (23) is comparatively shown in Figure 5.

Figure 5 shows that there are three peaks in frequency scope from 10–200 kHz. The peak frequency at Peak-1 on the theoretical curve is around 37.58 kHz, while the experimental frequency is 36.63 kHz. Hence, it is clearly observed that the calculated peak frequency is slightly larger than the measured one. By contrast, the magnitude of the theoretical curve at the Peak-1 is smaller than the experimental curve. The peak frequencies of Peak-2 and -3 are shifting towards the right compared with a peak frequency at Peak-1, but the magnitude of the two peaks are too small to be analyzed. To sum up, the similarity of Peak-1 on two curves can be regarded as the primary indicator when making a comparison between theoretically predicted and experimentally measured signals.

**Figure 6:**
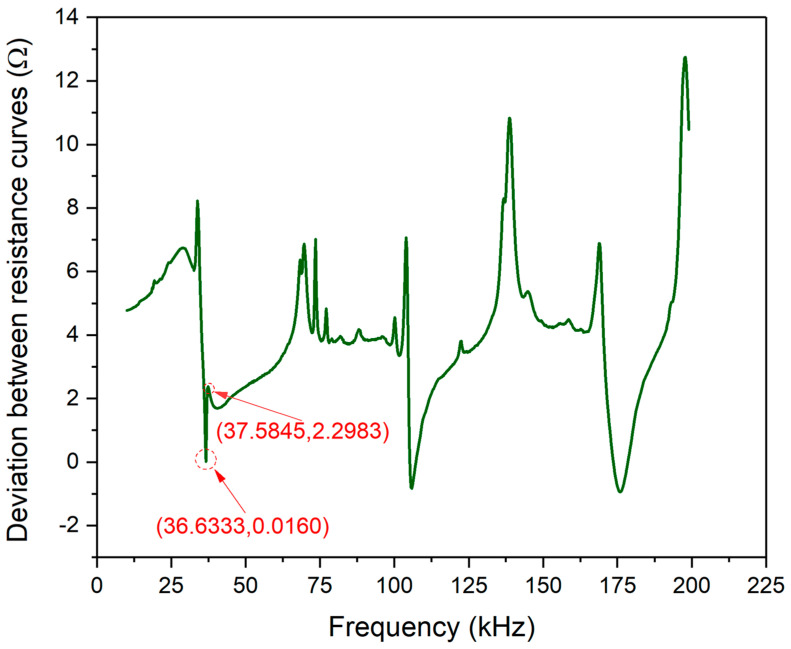
Resistance signal deviation between experimental and theoretical curves of PZT#1.

Figure 6 shows the relative deviation between the theoretical and experimental resistance curves of PZT#1. The signal between experimental and theoretical curves deviation is calculated by Equation (24):

(24) $$\eta_{RE}=\frac{x_{E}-x_{T}}{x_{E}}$$

$\eta_{RE}$ is a relative deviation indicator of experimental and theoretical signals. $x_{E}$ represents the raw signatures acquired in the experiment, while $x_{T}$ denotes the theoretically predicted signatures, respectively.

Although the magnitude of deviation changes with the frequency scope, it can be observed that the magnitude of the two curves is small enough on the whole. The range of vertical coordinates are confined up to 14 Ω, and the magnitude in the frequency scope where the Peak-1 situated is only from 0 to 8 Ω. Thus, the accuracy of the EMI model was proved preliminarily.

In order to further quantify the effectiveness and accuracy of the theoretical model for free PZT, an index of Pearson correlation coefficient (CC) was introduced to evaluate the similarity of signals between the experimental and theoretical curves. The expression of Pearson CC is defined by Equation (25).

(25) $$CC=\frac{1}{\sigma_{x}\sigma_{y}}\sum_{i=1}^{N}\left[\left(x-\bar{x}\right)\times \left(y-\bar{y}\right)\right]$$

where *x* and *y* represent experimental and theoretical data, respectively. $\bar{x}$ and $\bar{y}$ separately denote the mean value of *x* and *y*. $\sigma_{x}$ and $\sigma_{y}$ are the standard deviation of *x* and *y*. *i* is the data-counter and *N* is the total number of acquired data points.

**Figure 7:**
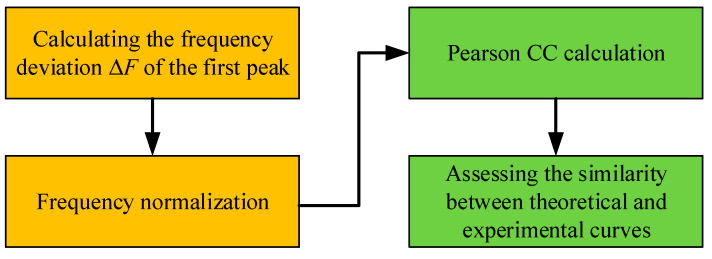
Flowchart of similarity assessment between theoretical and experimental curves.

Furthermore, a new method was presented in this paper on signal processing when assessing the similarity between theoretical and experimental curves in terms of CC index. The signal processing and similarity assessment between the two curves follows the procedure shown with a flow chart in Figure 7.

According to Equation (25) and Figure 7, the calculated Pearson CC based on the resistance signals of theoretical and experimental curves of PZT#1 can be obtained, which is 0.9290. The value of CC for PZT#1 convincingly demonstrated the EMI theoretical model’s effectiveness and accuracy. Moreover, the novel derivation route based on zero-stress assumptions on the boundary and longitudinal vibration was also proved to be reasonable.

**Figure 8:**
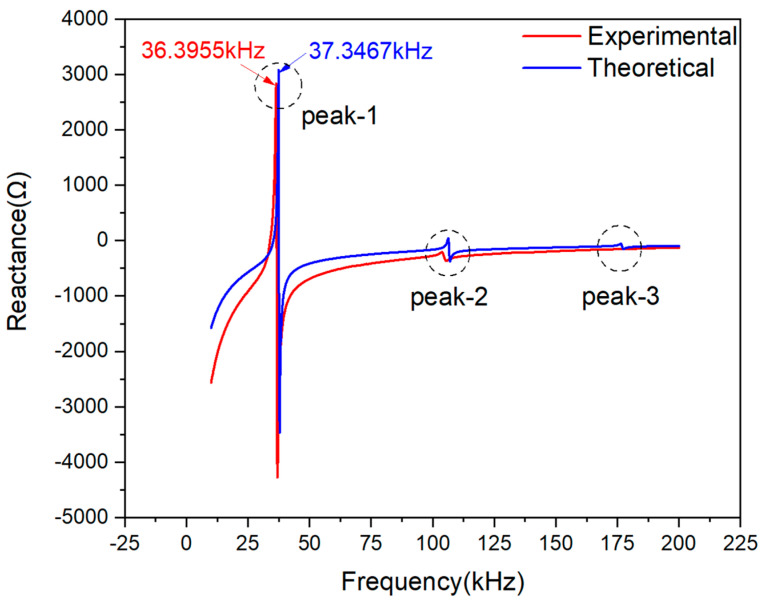
Experimental and theoretical reactance plots comparison of PZT#1.

Figure 8 shows good consistency between the theoretical and the experimental curves in their imaginary part. Three peaks were observed on reactance curves. The main peak marked with Peak-1 of experimental data is at the frequency point of 36.3955 kHz, while the theoretical one is 37.3467 kHz. Referring to the processing method on the basis of the resistance signals, the main peak frequency deviation between the two reactance curves at Peak-1 is 0.9512 kHz. Besides, there is also a deviation in signal magnitude at Peak-1 and -2, but the two peaks are too small in magnitude to be analyzed compared with Peak-1.

**Figure 9:**
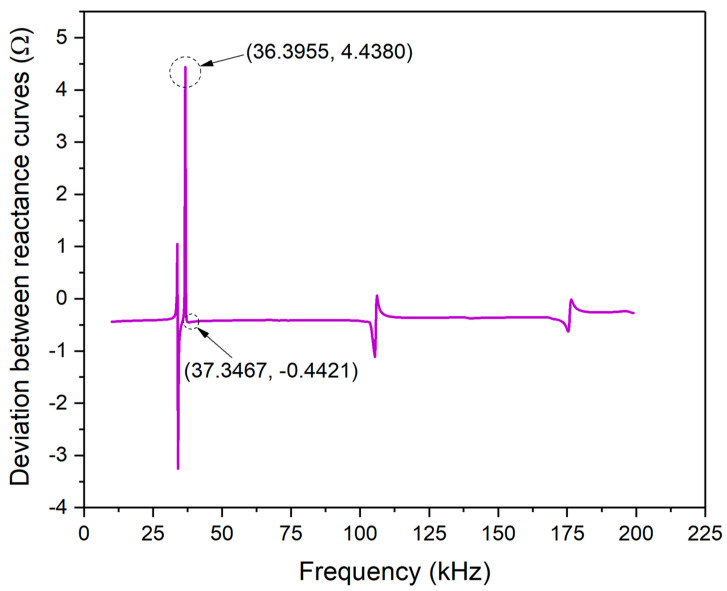
Reactance signal deviation between experimental and theoretical curves of PZT#1.

Figure 9 shows the deviation between the theoretical and experimental reactance curves of PZT#1. Despite the magnitude of deviation changing with the frequency scope, it can be observed that the magnitude of the two curves is small enough on the whole. The range of vertical coordinates within −4 to 5 Ω. In the frequency scope from 25 k to 50 kHz, one peak and one trough related to Figure 8 are marked, of which the magnitude is 4.4380 Ω and −0.4421 Ω. It demonstrates that there is good accuracy in predicting the reactance signal at the Peak-1.

To evaluate the similarity between the experimental and theoretically predicted curves, the Pearson CC was also calculated in terms of Equation (25) and Figure 7. The CC value based on measured and predicted reactance signatures is 0.9625. According to the CC index, it can be concluded that the EMI model for 1D free PZT derived with the novel assumptions in this paper shows satisfactory precision.

### 4.2. Comparison between Theoretical and Experimental Data of PZT#2

To further study the effectiveness of the EMI model presented in this paper, another experiment with PZT#2 was also conducted. Substituting the properties of PZT#2 listed in Table 1 into Equation (23), the theoretical plots can be obtained.

**Figure 10:**
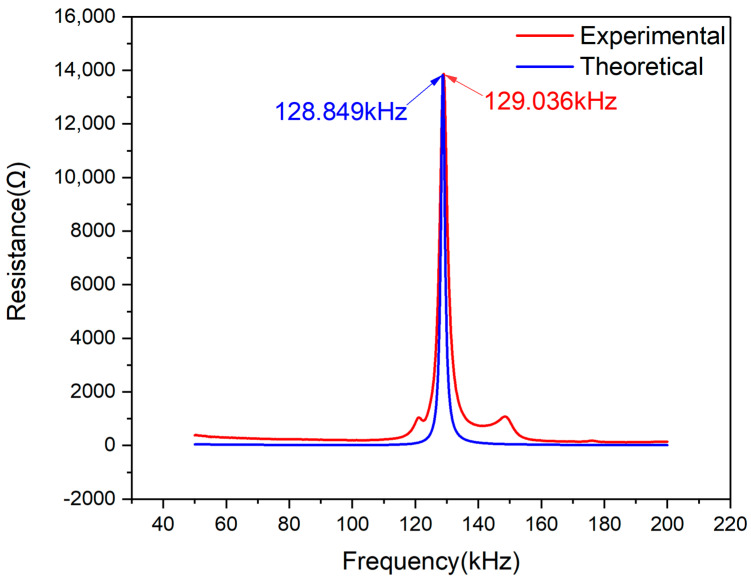
Experimental and theoretical resistance plots comparison of PZT#2.

In the frequency scope ranging from 50–200 kHz, the comparative resistance plots for PZT#2 are shown in Figure 10. It can be observed that there is only one peak in this frequency scope, both on the theoretical and experimental curves. By comparing the experimental and theoretical curves, one can find that the two curves’ peak frequencies are very close. On the other hand, it can also be clearly seen that the two curves’ magnitude is almost equal.

**Figure 11:**
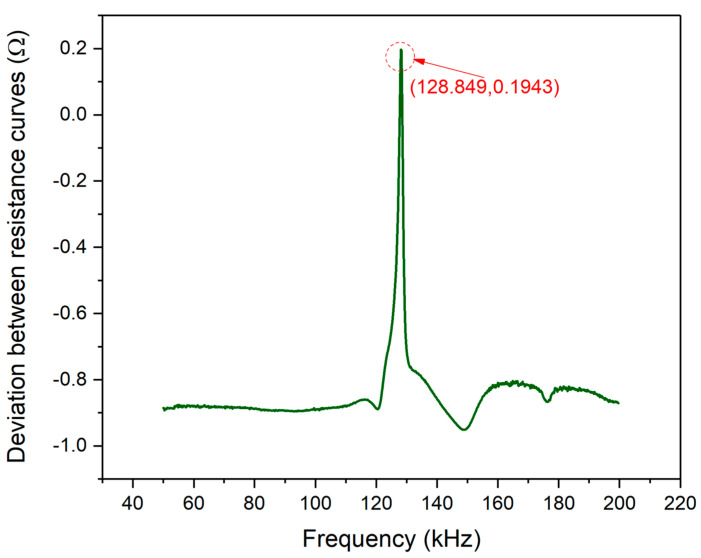
Resistance signal deviation between experimental and theoretical curves of PZT#2.

Figure 11 shows the deviation between the theoretical and experimental resistance curves of PZT#2. According to Equation (24), the deviation plot changing with the frequency scope was plotted and it can be observed that the magnitude of the two curves is tiny in magnitude: less than 1 Ω. The negative value of coordinates represents experimental values that are smaller than the theoretically predicted values. In the frequency scope from 120 k–140 kHz, one deviation peak related to Figure 10 is marked, whose magnitude is only 0.1943 Ω. Hence, the effectiveness of EMI model is further demonstrated.

Similar to the scenario of PZT#1, the Pearson CC of PZT#2 is also calculated, which is 0.9145. Hence, according to the value of Pearson CC, the effectiveness of the EMI model for PZT under free condition can be further validated.

**Figure 12:**
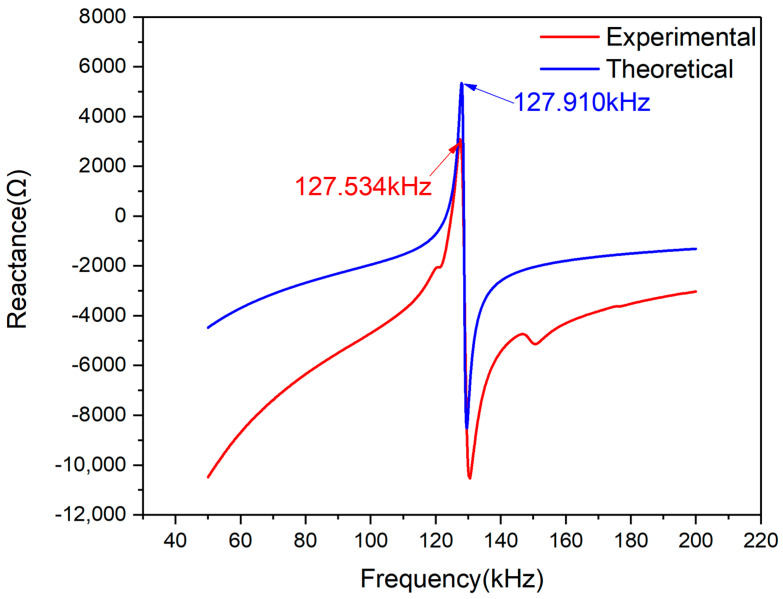
Experimental and theoretical reactance plots comparison of PZT#2.

The comparative reactance plots for PZT#2 are shown in Figure 12. It can be seen that there is one peak and a trough on the two curves. The peak frequencies of the two curves are very close, but differences in signal magnitude are apparent. The magnitude of the theoretical curve is larger than that of the experimental curve. On the contrary, the observed curve at the trough is lower than the theoretical one in magnitude. Furthermore, the trough frequency of theoretical curves is shifting towards the left compared with the experimental one, which means the predicted trough frequency is smaller than the actual value.

**Figure 13:**
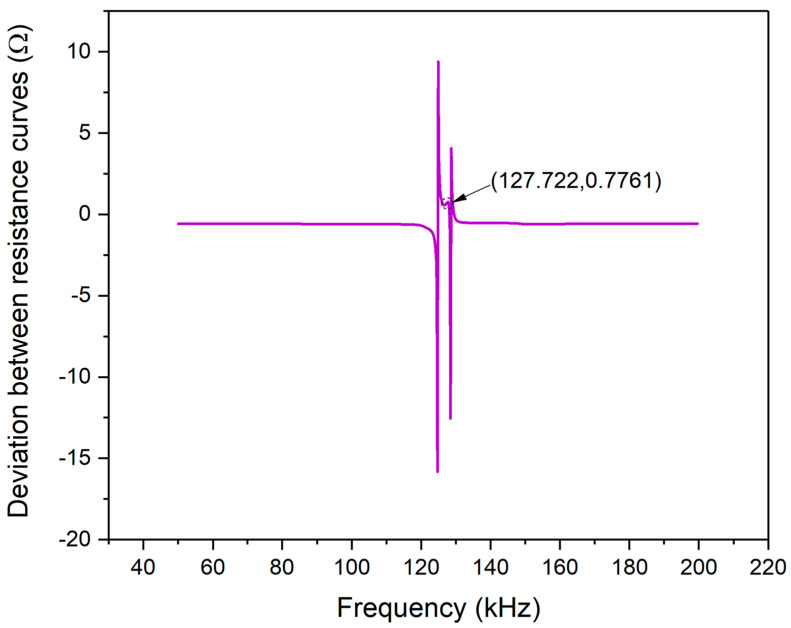
Reactance signal deviation between experimental and theoretical curves of PZT#2.

Figure 13 shows the deviation between the theoretical and experimental reactance curves of PZT#2. As can be seen from the figure, the two curves’ magnitude is confined to −20 to 10 Ω. The deviation between the experimental and theoretical curves at the frequency point of 127.722 kHz is only 0.7761 Ω. As mentioned above, the main peak is regarded as an indicator to evaluate the EMI model’s accuracy introduced in this paper. The comparative results demonstrate that the model has good precision in predicting the reactance signal from 50k to 200 kHz.

Using the method presented above to evaluate the similarity between the two curves shown in Figure 12, the Pearson CC index was also calculated. The CC index of PZT#2 based on reactance signatures in 50–200 kHz is 0.8863. According to the statistical theory, two data sets are highly relevant when their Pearson CC is from 0.8 to 1.0. In the light of CC value based on reactance signatures, theoretical and experimental plots are able to be regarded as having good similarity. Hence, the EMI model with a novel assumption and derivation process for free PZT condition can be further demonstrated with this investigation.

## 5. Conclusions

This paper presents an investigation on the EMI theoretical model derived with an assumption of zero-stress on the boundary of 1D thin-walled PZT that can vibrate freely in a longitudinal direction. To study the effectiveness of the EMI model proposed, experiments were conducted.

The EMI model is a universal one for the 1D PZT in a free state. It can be used for accurately predicting the resistance and reactance signatures in terms of piezoelectric ceramic material parameters and geometric size. Then the behavior of any 1D thin-walled PZT under free conditions is obtained. Hence, the modeling of a free PZT has potential for the screening and quality control before integrating it with monitored host structures, which could improve the cost-effectiveness in the field of PZT-based SHM adequately. The work presented in this paper mainly includes the following two aspects of novelties:

(1) A simplified derived process based on the assumption of zero-stress on the PZT boundary was proposed.
(2) A signal processing method of “frequency normalization” was proposed to evaluate the similarity of the EMI model by comparing frequency shifting and magnitude scaling to verify its effectiveness.

To evaluate the presented model’s effectiveness, a series of experiments were conducted by using piezoelectric ceramics, which were PZT#1 and #2. The theoretically predicted curves with the presented EMI model showed excellent accuracy when compared with the measured corresponding resistance and reactance signals. The comparative results between the theoretical and experimental of the real part and imaginary part of impedance signatures demonstrated the reasonability of the assumption and similarity assessment method proposed in this paper. Furthermore, this method has the potential application prospect of improving the cost-effectiveness and obtaining better monitoring results.

## Figures and Tables

**Table 1:** Properties and size of the PZT used in experiments.

Parameters	Units	PZT-5A	PZT-5A1
$s_{11}^{E}$	$10^{-12}\;m^{2}/N$	16.2	15.6
η	-	0.006	0.01
$\varepsilon_{33}^{T}/\varepsilon_{0}$	-	1920	1750
δ	-	0.006	0.01
$d_{31}$	$10^{-12}\;C/N$	−200	−160
ρ	$\mathrm{kg}/m^{3}$	7750	7700

## Abbreviations/Nomenclature

2*l*	Length of piezoelectric ceramics
*w*	Width of piezoelectric ceramics
*h*	Thickness of piezoelectric ceramics
$E_{3}$	Electric field in thickness direction
$s_{11}$	Compliance coefficient of piezoelectric ceramics
$u_{1}$	Displacement in longitudinal direction
$T_{1}$	Stress in longitudinal direction
ρ	Density of piezoelectric ceramics
κ	Wave number
ω	Angular frequency
$S_{1}$	Strain in longitudinal direction
$D_{3}$	Electric displacement
$d_{31}$	Piezoelectric strain constant
$\varepsilon_{33}^{T}$	Dielectric constant of piezoelectric ceramics
η	Mechanical loss factor of piezoelectric ceramics
δ	Dielectric loss factor of piezoelectric ceramics
*Y*	Admittance of piezoelectric ceramics
*Z*	Impedance of piezoelectric ceramics
